# How useful are clinical details in blunt trauma referrals for computed tomography of the abdomen?

**DOI:** 10.4102/sajr.v24i1.1837

**Published:** 2020-04-22

**Authors:** Kenneth B. Beviss-Challinor, Martin Kidd, Richard D. Pitcher

**Affiliations:** 1 Division of Radiodiagnosis, Department of Medical Imaging and Clinical Oncology, Faculty of Medicine and Health Sciences, Stellenbosch University, Cape Town, South Africa; 2 Centre for Statistical Consultation, Stellenbosch University, Stellenbosch, South Africa

**Keywords:** blunt trauma, tomography, X-ray computed, abdominal CT, justification, clinical content, electronic referral

## Abstract

**Background:**

The relevance of clinical data included in blunt trauma referrals for abdominal computed tomography (CT) is not known.

**Objectives:**

To analyse the clinical details provided on free-text request forms for abdominal CT following blunt trauma and assess their association with imaging evidence of intra-abdominal injury.

**Method:**

A single-institution, retrospective study of abdominal CT scans was performed for blunt trauma between 01 January and 31 March 2018. Computed tomography request forms were reviewed with their corresponding CT images. Clinical details provided and scan findings were captured systematically. The relationship between individual clinical features and CT evidence of abdominal injury was tested using one-way cross tabulation and Fisher’s exact test.

**Results:**

One hundred thirty-nine studies met inclusion criteria. A wide range of clinical details was communicated. Only clinical abdominal examination findings (*p* = 0.05), macroscopic haematuria (*p* < 0.01), pelvic fracture or hip dislocation (*p* = 0.04) and positive focused assessment with sonography in trauma (*p* < 0.01) demonstrated an associated trend with abdominal injury.

**Conclusion:**

Key abdominal examination and basic imaging findings remain essential clinical details for the appropriate evaluation of CT abdomen requests in the setting of blunt trauma. Methods to improve consistent communication of relevant clinical details are likely to be of value.

## Introduction

Trauma remains a major global public health problem and is the leading cause of hospitalisation, long-term disability and death in persons aged less than 40 years.^1^ Blunt trauma accounts for the majority of injuries.^2^

Approximately 15% of patients admitted in level-1 trauma centres have abdominal injuries.^3^ Clinical evaluation of blunt abdominal trauma has low sensitivity and specificity for injured organs. In neurologically intact, blunt trauma patients, trauma surgeons miss almost half of all abdominal injuries prior to imaging.^4^ Furthermore, missed injuries are more frequent in severe trauma, whilst in patients with decreased level of consciousness, injuries may be occult.^5^ Computed tomography (CT) has thus emerged as a definitive investigation for blunt abdominal injuries. However, there are concerns about its overutilisation, thereby exposing patients to unnecessary ionising radiation and inflating healthcare costs.^6,7^

South Africa has no local guidelines informing patient selection for CT abdomen in the setting of blunt trauma. International studies in this domain so far have been confounded by wide disparity in study design and terminology.^8,9,10,11^ Nonetheless, most guidelines recommend whole-body CT for major blunt trauma in adult patients Injury Severity Score > 15.^12,13,14,15^ However, the National Institute for Health and Care Excellence (NICE) in the UK acknowledges low-quality observational studies that inform such guidelines. Additionally, although various clinical decision tools have been developed in an attempt to identify patients at a very low risk of abdominal injury, and thus not warranting further investigation, uptake of such guidelines has been very limited.^16,17,18^

Justification is the decision-making process whereby the perceived benefits and potential risks of an examination are considered. Radiologists are responsible for the justification of medical exposure to ionising radiation, including trauma-related CT scans.^19^ To this end, referring clinicians are obliged to provide radiologists with sufficient clinical information to assess the merits of each investigation.

Many radiology departments utilise free-text referrals to convey information from clinician to radiologist. This communication is integral for optimising patient care and healthcare resources by ensuring that every scan is warranted, appropriately performed and reported.

The American College of Radiology stipulates that referrals should include relevant clinical information, a working diagnosis, pertinent signs and symptoms, as well as a specific clinical question.^20^ The Royal College of Radiologists suggests that clinical information might include medical symptoms or signs pointing to a particular diagnosis or range of diagnoses. The radiologist reporting the examination should understand the ‘explicit and implied information’ contained in the clinical details, appreciating their relevance and diagnostic importance when interpreting the imaging study.^21^ The quality of clinical detail on the referral should thus enhance both patient selection and the reporting of studies.^22^ However, a key challenge confronting the radiologist contemplating CT requests for trauma patients is that the value of any specific clinical detail provided in predicting abdominal injury is unknown to both referring clinician and radiologist.

Without insights into the association between request content and CT findings, the free text request is potentially arbitrary. Establishing a link between clinical request and CT findings would enhance the justification process.

We, therefore, aimed to describe the specific clinical details that clinicians elect to communicate and assess whether there is any association with imaging evidence of abdominal injury.

## Research methods and design

This was a retrospective analysis of the free-text request forms and corresponding images of all abdominal CT scans performed for blunt trauma from 01 January 2018 through 31 March 2018 at Tygerberg Hospital (TBH). Tygerberg Hospital is a 1386-bed tertiary-level, public sector facility in Cape Town, South Africa. It is the main teaching hospital of the Faculty of Medicine and Health Sciences of Stellenbosch University and has a level-1 equivalent trauma unit, which manages approximately 24 000 cases annually. Tygerberg Hospital has a digital, filmless and paperless imaging environment, in which all examinations are requested electronically. The electronic workflow requires formal approval of the radiologist for all imaging investigations except plain radiographs, and precludes scheduling of special examinations prior to the radiologist’s justification.

A systematic search of institutional radiology information system (RIS) was undertaken for initial abdominal CT scans of all adult patients (aged > 18 years) investigated for blunt trauma during the review period. Studies were excluded if CT scans were performed following laparotomy, as repeat scans, or without intravenous contrast.

All clinical data included on the free-text electronic CT requests were systematically captured on a customised Microsoft Excel spreadsheet and stratified by patient demographics, clinical history, vital signs and haemodynamic status, abdominal and extra-abdominal examination findings, side-room investigations, laboratory test results, and baseline imaging features.

Definitions for positive abdominal and extra-abdominal examination findings were adapted from previous studies evaluating abdominal examination in blunt trauma.^5,23^ A positive abdominal examination included any specification of abdominal bruising or abrasions, abdominal pain, tenderness or distension, low back pain, macroscopic haematuria or bruised chest. A positive extra-abdominal examination included specification of suspected injury to the head, cervical spine, thorax, thoracolumbar spine, pelvic fracture or hip dislocation, or long bone fractures.

All CT scans were re-reported by the lead investigator (K.B.C.), a radiology registrar with 4 years of experience. Any discrepancies with the final radiology report were resolved by consensus in consultation with the same consultant radiologist who authorised the report. Computed tomography findings were stratified by abdominal organ, utilising the respective American Association for the Surgery of Trauma (AAST) classifications for solid organ injury.^24^ Extra-abdominal injuries were also captured (Table 1).

**TABLE 1:** Criteria defining a computed tomography diagnosis of abdominal injury and/or extra-abdominal injury.

CT diagnosis of abdominal injury	CT diagnosis of extra-abdominal injury
Intraperitoneal free fluid†	Lower rib fracture
Intra- or retroperitoneal free air	Diaphragm injury
Solid organ injury‡	Thoracolumbar spine injury§
Ureter or urinary bladder injury	Isolated transverse process fractures
Bowel or mesenteric injury	Pelvic or proximal femur fractures or hip dislocation
Vascular injury	-

CT, computed tomography.

† , Excluded minimal free fluid in absence of other evidence of abdominal injury;

‡ , Liver, spleen, pancreas, kidney or adrenal injury;

§ , Excluded isolated transverse process fractures.

Relationships between variables on electronic request and CT diagnosis of abdominal injury were tested using cross tabulation and Fisher’s exact test. Age differences were tested using one-way analysis of variance.

### Ethical consideration

This was a retrospective study. Confidentiality was maintained at all times. The study was approved by the Stellenbosch Research Ethics Committee (Reference No. S18/07/142).

## Results

One hundred thirty-nine patients (*n* = 139), with a mean age of 37 years (male: *n* = 110, 79%) were included in the analysis. In most patients (*n* = 86; 62%), the abdominal study was performed in conjunction with CT of other anatomical regions; in 28 patients (20%) it was a part of whole-body CT and in 25 (18%), CT was limited to the abdomen.

### Request form content

Request form information is presented in Table 2. A total of 976 diverse free-text clinical details were provided on 139 request forms, reflecting an average of seven details per referral. All referrals (*n* = 139, 100%) provided at least one abdominal (*n* = 95, 68%) or extra-abdominal (*n* = 127, 91%) clinical examination finding; 134 (96%) included a detail on clinical history; 70 (50%) reported side-room investigations; 45 (32%) reported baseline imaging results and 14 (10%) gave laboratory findings. No referral reflected detail in all categories.

**TABLE 2:** Frequency of request form detail (*N* = 139).

Section	Item	Frequency	%
History	Any entry	134	96
	Total entries	171	-
	Mechanism of injury	132	95
Vital signs or haemodynamic status	Any entry	33	24
	Total entries	35	-
Abdominal examination	Any entry	95	68
	Total entries	209	-
	Unreliable	27	19
Extra-abdominal examination	Any entry	127	91
	Total entries	324	-
	GCS/mental status†	97	70
Side-rooms	Any entry‡	70	50
	Total entries	119	-
Lab tests	Any entry	14	10
	Total entries	14	-
Imaging	Any entry	45	32
	Total entries	104	-
	FAST§	24	17

GCS, Glasgow Coma Scale; FAST, focused assessment with sonography in trauma.

† , Any mention of GCS or mental status, whether quantified or not;

‡ , Urinalysis was ‘pending’ in one request form;

§ , FAST was ‘unavailable’ in four requests forms.

Mechanism of injury (*n* = 132; 95%) was specified in the majority of cases, the Glasgow Coma Scale or mental status (*n* = 97; 70%) was reported in just over two-thirds of patients, and microscopic haematuria (*n* = 69; 50%) in approximately half the referrals. Of note, abdominal examination was deemed ‘unreliable’ in almost one-fifth of cases (*n* = 27; 19%) because of the patient’s decreased level of consciousness. Almost one-quarter (*n* = 33; 24%) recorded vital signs or haemodynamic status, and less than one-fifth referred to focused assessment with sonography in trauma (FAST; *n* = 24; 17%).

### Computed tomography findings

Abdominal injury was diagnosed in approximately one-quarter of patients (*n* = 36; 26%), most of whom had solid organ injury (*n* = 34; 24%). There was no AAST grade 5 injury. There were six grade 4 (4%) and 35 (25%) grade 3 or less AAST injuries documented. Grade 4 injuries involved the liver (*n* = 3), spleen (*n* = 2) and kidney (*n* = 1), all having positive abdominal examinations. The five non-solid organ injuries involved the bladder (*n* = 2), abdominal vasculature (*n* = 2) and bowel (*n* = 1) – all were associated with solid organ injury and had positive abdominal examination, X-ray or FAST findings (Table 3).

**TABLE 3:** Computed tomography abdomen findings.

Organ injured	Subcategory	Grading	Frequency (*N* = 139)	%
Any abdominal injury†	-	-	36	26
Free fluid	-	-	30	22
Free air	-	-	3	2
Solid organ	-	-	34	24
	Liver	-	25	20
		Liver, grade 5	0	
		Liver, grade 4	3	2
		Liver, grade 3	10	7
		Liver, grade 2	9	6
		Liver, grade 1	3	2
	Spleen	-	8	6
		Spleen, grade 5	0	
		Spleen, grade 4	2	1
		Spleen, grade 3	3	2
		Spleen, grade 2	1	1
		Spleen, grade 1	2	1
	Kidney	-	8	6
		Kidney, grade 5	0	
		Kidney, grade 4	1	1
		Kidney, grade 3	5	4
		Kidney, grade 2	2	1
		Kidney, grade 1	0	
Non-solid organ	Ureter	-	0	0
	Bladder	-	2	1
	Bowel	-	1	1
	Mesenteric	-	0	0
	Vascular	-	2	1
	-	Vascular contrast extravasation	1	1

† , Total patients with any single injury or a combination of injuries.

### Association between electronic request and abdominal injury

The association between request form details and CT evidence of abdominal injury is summarised in Table 4.

**TABLE 4:** Association between request form detail and computed tomography evidence of abdominal injury.

Section	Item	Frequency		%	Abdominal injury present		%	p*
		*n*	*N*		*n*	*N*		
History	**Mechanism**	132	139	95	32	132	24	0.08
	Vehicle accidents (MVA & PVA)	85	132	64	23	85	27	-
	Assault	22	132	17	7	22	32	-
	Other	25	132	19	2	25	8	-
Examination	**Vital signs**†	28	139	20	6	28	21	0.06
	Normal	10	28	36	0	10	0	-
	Abnormal	18	28	64	6	18	33	-
	**Abdominal exam**§	75	139	54	26	75	35	0.05
	Positive	66	75	88	23	66	35	-
	Negative	9	75	12	0	9	0	-
	**Macroscopic haematuria**	66	139	47	23	66	35	< 0.01
	Present	17	66	26	11	17	65	-
	Absent	49	66	74	12	49	24	-
	**GCS¶**	92	139	66	23	92	25	0.79
	14–15	28	92	30	6	28	21	-
	≤ 13	64	92	70	17	64	27	-
	**Extra-abdominal injury**¶¶	117	139	84	52	117	44	-
	Head	77	117	66	19	77	25	0.66
	Facial	17	117	15	4	17	24	1
	Cervical spine	7	117	6	2	7	29	1
	Thoracic	26	117	22	8	26	31	0.62
	Dorsal spine	9	117	8	1	9	11	0.44
	Lumbar spine	7	117	6	0	7	0	0.19
	Pelvic fracture or hip dislocation	25	117	21	11	25	44	0.04
	Long bone fracture	17	117	15	7	17	41	0.15
Side-rooms	**Microhaematuria**	69	139	50	14	69	20	0.58
	Present	64	69	93	14	64	22	-
	Absent	5	69	7	0	5	0	-
Imaging	**FAST**	20	139	14	7	20	35	< 0.01
	Positive	11	20	55	7	11	64	-
	Negative	9	20	45	0	9	0	-

MVA, motor vehicle accident; PVA, pedestrian vehicle accident; GCS, Glasgow Coma Scale; FAST, focused assessment with sonography in trauma.

* , Fisher’s exact test;

† , Analysis includes all cases classifiable as normal or abnormal;

§ , Analysis includes all cases classifiable as positive or negative;

¶ , Analysis includes all cases where GCS was quantified;

¶¶ , Analysis excludes GCS/mental status, intoxication status and intubation status.

Referral details trending towards association with abdominal injury were positive abdominal examination (*p* = 0.05), macroscopic haematuria (*p* < 0.01), pelvic fracture or hip dislocation (*p* = 0.04) and positive FAST (*p* < 0.01). Negative abdominal examination, absence of macroscopic haematuria and negative FAST were negatively associated with abdominal injury.

Thus, only 186/976 (19%) of all clinical details on request forms were associated with abdominal injury.

## Discussion

There is a wealth of literature investigating the value of various signs and symptoms, side-room tests and imaging investigations in predicting the probability of abdominal injury. In a systematic review, Nishijima et al.^5^ concluded that bedside ultrasonography had the highest accuracy, that a combination of clinical findings were likely to be most useful to select patients who were unlikely to benefit from further evaluation, but that the ideal combination was yet to be determined. To our knowledge, this is the first study to investigate whether these are reliably reflected in the free-text request form.

Our study had a number of key findings. Firstly, only 19% of clinical details reflected on free-text referrals at our institution were associated with imaging evidence of abdominal injury. This calls into question the role of such referrals and suggests there is room for substantial improvement in this domain.

Secondly, we reaffirmed the pivotal role of meticulous clinical abdominal examination in the setting of blunt trauma. Of note, only 54% of patients undergoing abdominal CT had mention of abdominal examination findings interpretable as positive or negative. A potential explanation for this less than comprehensive documentation of abdominal findings is that guideline-driven imaging protocols frequently recommend abdominal CT to rule out occult abdominal injuries in patients with head injuries, citing that clinical abdominal examination is likely to be unreliable in this setting. This could lead less experienced clinicians to assume that abdominal examination findings are of limited value. Our study suggests that abdominal examination findings form a relevant component of referral and should be provided, whether positive or negative. A normal abdominal examination is predictive of the absence of abdominal injury.

Thirdly, our study suggests that FAST could play a role in the reduction of abdominal CT utilisation in the blunt trauma setting, in line with findings of Sheng et al.^25^ Efforts should be made to expand the use of FAST. This would involve developing appropriate skills amongst clinicians. Focused assessment with sonography in trauma training programmes are increasingly available, and ultrasound machines are becoming more affordable and portable. Any unit with a CT scanner should be equipped with basic ultrasound equipment.

We found no evidence to support the provision of qualitative or quantitative information about microscopic haematuria, consistent with studies demonstrating its low value.^26,27^ The relatively high frequency (50%) of documentation on electronic requests could be because of the speed and ease of the test during the trauma admission procedure. Clinicians equivocating about the need of CT could include microscopic haematuria as a further motivation for requesting CT, particularly in the absence of other compelling indications. Our findings suggest that CT abdomen for isolated microscopic haematuria, in the absence of any other finding, is not justified.

Although free text electronic requests permit clinical information to be communicated without constraint, a major limitation is that provision of relevant content remains voluntary. Methods of improving the consistent provision of relevant clinical content on imaging requests are likely to strengthen the evidence base of this information and facilitate an optimal justification process. There may be a role of a drop-down menu-style electronic request, thus obliging referring clinicians to provide relevant information and restricting the communication of irrelevant information.

There are limitations in the statistical analysis of free text referrals. The retrospective design meant that parameters were not specified in every case, thereby preventing determination of their diagnostic sensitivities and specificities. Prospective studies are needed to analyse systematically the diagnostic value of various contents on electronic request. Furthermore, we only conducted a univariate analysis. Given the complex clinical variables in trauma, a multivariate analysis controlling for confounding variables would represent a superior analysis. The capture of data pertaining to imaging tests performed prior to CT was limited in that it was occasionally impossible to determine whether findings had been diagnosed on examination or on imaging tests.

The strengths of the study included the availability of comprehensive RIS data and ability to enrol consecutive patients with little exclusion.

## Conclusion

Key abdominal examination and basic imaging findings remain essential clinical details for the appropriate evaluation of CT abdomen requests in the setting of blunt trauma. Requests specifying a positive abdominal examination, macroscopic haematuria, pelvic fracture or hip dislocation, or positive FAST are associated with CT evidence of abdominal injury. Methods to improve consistent communication of relevant clinical details are likely to be of value.
